# COVID-19 impact on urology practice: A possible dilemma of misdiagnosis

**DOI:** 10.1080/2090598X.2020.1755947

**Published:** 2020-04-21

**Authors:** Elsayed Desouky

**Affiliations:** Department of Urology, Wexham Park NHS Hospital, Berkshire, UK; Department of Urology, Alexandria Main University Hospital, Alexandria, Egypt, sayedurology@hotmail.com

Coronavirus disease 2019 (COVID-19) is the infectious disease caused by the most recently discovered coronavirus, which began as an outbreak in December 2019 in Wuhan, China. On 11 March 2020, the WHO announced COVID-19 as a pandemic, with Europe being its epicentre. The worst hit country in Europe was Italy, where they reported the most cases and fatalities after China [1].

On 1 April 2020, the Middle East represented an about 9–10% regional burden of the global caseload, with a crude number of ~70 000 confirmed cases; 779 in Egypt, 1853 in Saudi Arabia, 871 in the Emirates, 740 in Iraq, 506 in Lebanon, 536 in Kuwait, 482 in Tunisia, and 316 in Jordan [1]. At the same time point, the reported number of cases in the UK was 40 806 [2], while the USA reported the world’s highest number of patients, with >300 000 confirmed cases [1].

Despite the fact that reported numbers in the Middle East and North Africa are not as massive as those in Europe or the USA, the WHO has been reporting increasing numbers of cases that have tested positive for COVID-19 [1]. This is particularly alarming for the Arab World considering the limited resources available for healthcare facilities in most of the Middle Eastern countries compared to Europe or the USA.

Middle East Respiratory Syndrome (MERS), first identified in Saudi Arabia in 2012, has a higher mortality rate than COVID-19 (35% vs 3.4%). However, it is important to note that although the resulting case-fatality percentage of COVID-19 is relatively smaller, the disease has already killed more than MERS in absolute values [1].

COVID-19 has a higher transmissibility, which can lead to a sharp surge in the curve of overall infected cases within a short period of time. This can lead to enormous pressure on hospitals and subsequently ‘crashing’ healthcare systems.

Arab countries have been taking drastic measures to face this pandemic as per WHO guidance. Public health education campaigns, emphasising the importance of frequent hand washing and social distancing, have been broadcast on television, radio stations and social media platforms. In addition, health authorities have been managing to increase hospital capacities and resources in order to contain the crisis [1].

## Impact on urology service

From a urological point of view, there is no direct pathological effect of COVID-19 on the urinary tract [3]. However, urologists are still getting involved during this pandemic in the fossllowing aspects:

- The massive impact on healthcare systems needs a proper plan to face the anticipated surge in the numbers of patients; hence, the need to reduce the level of activity in surgical specialties including urology. This stepping down approach is necessary to achieve the following:
  - To follow infection control guidance minimising the number of patients attending hospital premises.
  - To increase bed capacity for any possible sharp rise in COVID-19 admissions.
  - To make all necessary equipment available, e.g. masks and ventilators.
  - To free as many staff members as possible to support healthcare demands, to attend refresher courses and to be trained for other alternative roles.
- Dealing with COVID-19 patients who need a urological consultation implies a risk of infection. A task as simple as insertion of a urinary catheter can expose the urologist to the hazard of droplet infection when the patient is coughing.

No viral load has been detected in urine samples, but it has been found in stools. The WHO stated that the risk of catching COVID-19 from the faeces of an infected person appears to be low. While initial investigations suggest the virus may be present in faeces in some cases, spread through this route is not a main feature of the outbreak [1]. This applies mainly to faeco–oral route of transmission, but raises concerns about the hazard of droplet exposure or splashing upon performing DRE and TRUS.

According to BAUS updated guidance for cancer diagnostics and treatment, no recommendations were made against DRE or TRUS [4], but urologists need to consider appropriate personal protective equipment (PPE) on attempting such diagnostics.

- Another risk posed by the current situation is during the surge phase of the crisis when urologists are asked to take an alternative role as physicians attending COVID-19 wards, supporting patients care in a very unfamiliar environment.
- Physical and mental stress during such hard times can be overwhelming and must be taken into consideration.

## Triaging patients

During the COVID-19 pandemic, there will be a reduced elective service provision for our patients. Urologists need to adopt a pragmatic approach for patient management. This approach may need to deviate from the internationally accepted standard of care and our daily practice.

The BAUS has published a valuable guidance for management of urological patients during the pandemic [4]. They have taken into consideration classifying certain procedures into low or high risk based on intensive care unit capabilities in face of high demand for ventilators to support patients with COVID-19. This guidance is simplified in Table 1.

**Table 1.** Prioritisation of urological procedures.

First to be cancelled	Second to be cancelled	Last to be cancelled	Emergency cases
Day surgery, e.g. varicocoele/hydrocoele	Cystectomy (low risk)	Cystectomy (high risk)	Testicular torsion
Benign nephrectomy	TURBT (low risk)	TURBT (high risk)	Obstructed infected kidney
Andrology	Radical prostatectomy	Radical nephrectomy	Abscess/gangrene
Functional/reconstructive surgery	Nephroureterectomy (low risk)	Nephroureterectomy (high risk)	
PCNL		Inguinal orchidectomy	
TURP, HoLEP and other procedures for BPH		Ureteroscopy with ureteric stone/stented patient	

HoLEP: holmium laser enucleation of the prostate; PCNL: percutaneous nephrolithotomy; TURBT: transurethral resection of bladder tumour.

The Royal College of Surgeons (RCS) published guidelines on 26 March 2020 for surgeons working during the pandemic, stating that acute patients are the priority. For emergency patients as well as any patient prioritised to undergo urgent planned procedures, surgeons need to check the COVID-19 status via history, testing, CT chest or at least a chest X-ray. As well, any patient undergoing a CT scan of the abdomen must have a CT chest done [5]. The RCS concluded that this pandemic represents a challenge that could generate a large number of patients in a short period of time so how to manage this, is still evolving [5].

## Risk of misdiagnosis

During this pandemic, urologists need to be aware of the possible overlap in patients’ presentation that can lead to misdiagnosis.

Fever was identified in 43.8% of patients with COVID-19 at presentation [3]. Some patients can present to Accident and Emergency with fever together with having a long-term catheter, a nephrostomy tube or a ureteric stent already in place. These patients can be misdiagnosed as urosepsis instead of COVID-19 or vice versa. A high index of suspicion must be kept in mind and these patients need to be managed in isolation until a definite diagnosis is confirmed.

In this case, laboratory results can be helpful in giving physicians a clue to guide their clinical judgment. Checking the full blood count is crucial, as lymphopenia has been found in >80% of patients with COVID-19 [3], opposite to the leucocytosis with neutrophilia associated with bacterial sepsis.

A serum ferritin test can provide another clue as to a COVID-19 diagnosis. Elevated serum ferritin is an early marker for ‘cytokine storm’, which has been proposed as a pathophysiological mechanism for the severe morbidity associated with COVID-19 [6].

According to the European Association of Urology (EAU) Guidelines, procalcitonin is a raised inflammatory marker in bacterial sepsis and can be useful to differentiate it from severe viral infections or other severe inflammatory reaction of non-infectious origin [7]. COVID-19 does not seem to increase the procalcitonin level. In the largest series, procalcitonin levels were <0.5 ng/mL in 95% of patients with COVID-19 [3].

On the other hand, fever developed in ~88.7% of patients with COVID-19 after hospitalisation [3]. This is a challenging situation when a patient under our care for any urological reason starts to temperature ‘spike’ and later is diagnosed with COVID-19. In such cases, the National Institute for Health and Care Excellence (NICE) guidelines [8] recommends the following:

- If COVID-19 is diagnosed in someone not isolated from admission or presentation, healthcare workers (HCWs) who come into contact with a patient with COVID-19 while not wearing PPE can remain at work. This is because in most instances this will be a short-lived exposure, unlike exposure in a household setting that is ongoing.
- HCWs should not attend work if they develop symptoms while at home (off-duty), and notify their line manager immediately self-isolate, and immediately inform their line manager if symptoms develop while at work.

These clinical scenarios highlight the importance of the awareness of COVID-19 clinical presentations by urologists in order to avoid the risk of misdiagnosing our patients, as well as not to spread the infection amongst others.

## Conclusion

The COVID-19 pandemic is a global unprecedented event posing a major impact on all healthcare systems. As urologists, we need to be well prepared ahead of time to face such a challenge in these extraordinary circumstances. Reduced elective service provision, the need to triage patients, and the possible overlap of symptoms and signs, all are examples of the impact of COVID-19 on our daily practice. Guidelines are continuously changing as the situation evolves and this needs utmost readiness and flexibility to maintain safety of our patients and healthcare professionals in keeping with good medical practice.
